# Thrombocytopenia according to antiretroviral drug combinations, viremia and CD4 lymphocytes among HIV-infected patients in Cameroon: a snapshot from the City of Yaoundé

**DOI:** 10.1186/s13104-019-4664-7

**Published:** 2019-09-26

**Authors:** Alex Durand Nka, Samuel Martin Sosso, Joseph Fokam, Yagai Bouba, Georges Teto, Rachel Simo Rachel, Aline Tiga, Junie Yimga, Elias Nchiwan Nukenine, Aubin Joseph Nanfack, Désiré Takou, Zélateur Aroga, Vittorio Colizzi, Alexis Ndjolo

**Affiliations:** 1 “Chantal BIYA” International Reference Centre for Research on HIV/AIDS Prevention and Management, (CIRCB), Yaoundé, Cameroon; 2 Faculty of Sciences, University of Ngaoundéré, Ngaoundéré, Cameroon; 3 Faculty of Medicine and Biomedical Sciences, University of Yaoundé I, Yaoundé, Cameroon; 4 Ngaoundéré Presbyterian Hospital, Ngaoundéré, Cameroon; 5 University of Rome “Tor Vergata”, Rome, Italy

**Keywords:** HIV, Thrombocytopenia, Antiretroviral triple therapy, Viral load, CD4, Cameroon

## Abstract

**Objective:**

Thrombocytopenia is an abnormal decrease in blood platelets, which can affect the prognosis of people living with HIV (PLHIV). In order to assess the burden of this haematological disorder, we evaluated the frequency of thrombocytopenia according to antiretroviral drug combinations, viremia and the immune status of PLHIV.

**Results:**

A cross-sectional and analytical study was conducted from June to November 2016 among 310 PLHIV at the “Chantal BIYA” International Reference Centre, Yaoundé, Cameroon. Overall rate of thrombocytopenia was 19.0% (59/310). The rate of thrombocytopenia was 64.6% (42/65) versus 6.9% (17/245) in ART-naïve versus ART-treated patients respectively, p < 0.0001. Following viral load, rate of thrombocytopenia was 15.8% (20/130) in those with undetectable viral load, and 34.1% (27/79) with viral loads > 3 log_10_ RNA/ml (p = 0.03). As concerns CD4-count, rate of thrombocytopenia was 16.2% (42/259) in those with ≥ 200 CD4/mm^3^ versus 33.3% (17/51) with < 200 CD4/mm^3^ (p = 0.0003). After adjusting for sex, ART, viral load and CD4, Viral load and ART exposure were significantly associated with decreased risk of thrombocytopenia (p < 0.05). Thrombocytopenia occurs especially among ART-naïve, high viremia and severe immune-compromised patients. Interestingly, ART coverage appears as an independent factor in preventing the occurrence of thrombocytopenia.

## Introduction

Thrombocytopenia is a condition characterized by an abnormally low number of platelets in the blood. In clinical practice, this refers to a number of platelets less than 150,000 per mm^3^ of blood [1]. In the context of HIV infection, events of thrombocytopenia are likely due to either the drug-induced adverse events or the infection itself [2]. Thrombocytopenia may therefore be suggestive of an increase in viremia, an alteration of the immune system and could also be due to the type of antiretroviral therapy (ART), effects of opportunistic infections and malignancies [2].

In the western world, monitoring of HIV-infected patients is focused on regular measurements of viral load and CD4 lymphocytes, and this approach is becoming gradually accessible in resource-limited settings (RLS), including sub-Saharan Africa (SSA) [3]. With limited evidence on possible correlations/associations between thrombocytopenia and ART-therapy in SSA settings, it becomes relevant to generate findings that would guide on the occurrence of thrombocytopenia following exposure or non-exposure to ART, following type of ARV drug combinations, and following the viral dynamics and immune status of patients in SSA countries [4].

The aim of our study was to assess the burden of thrombocytopenia according to antiretroviral therapy (ART), viremia and CD4-lymphocytes count of PLHIV.

## Main text

### Methods

#### Study design and setting

A cross-sectional and analytical study was carried out among PLHIV in Yaoundé, Cameroon, monitored at the “Chantal BIYA” International Reference Center for research on HIV/AIDS prevention and management (CIRCB). CIRCB is a government institute of the Ministry of Public Health, in charge of research and reference clinical monitoring of HIV-infected patients, with participation in external quality assurance programs for HIV screening/diagnosis, viral load measurements, CD4 count, as well as biochemistry and haematological analysis (http://circb.cm/btc_circb/web/).

Following a consecutive sampling, a total of 310 participants were enrolled, based on the following criteria: (a) HIV-positive confirmed using the national algorithm in Cameroon; (b) providing of informed consent/assent for enrolment in the study; (c) documented treatment history. Non-inclusion was based on: events of pregnancy; co-infection with malaria; viral hepatitis B and C; or those who underwent blood transfusion during the past 3 months.

#### Laboratory procedures

At the medical units of CIRCB, the study information sheets were provided to each potential eligible patient and written informed consent/assent was obtain from every participant. Sociodemographic data were obtained by data abstraction on a standard questionnaire for each participant. Clinical and therapeutic data were obtained from the medical records of each participant registered in the CIRCB database.

Briefly, 4 mL of whole blood was collected in a dry tube (for serological analysis) and 2 tubes of 4 mL of whole blood were collected in Ethylenediamine tetra acetic acid (EDTA) containing-tubes. The serum obtained from blood in the dry tubes was immediately used for carrying out the serological tests (HIV, Hepatitis B and C) by solid phase immunochromatography. Centrifugation of one EDTA tube was performed to obtain plasma which was then separated in two (2) 700 μL aliquots and stored at  − 20 °C before analysis for viral load by Real Time PCR on the ABBOTT m2000RT platform as per the manufacturer’s instructions (http://www.abbottmolecular.com/products/infectious-diseases/realtime-pcr/hiv-1-assay); the second EDTA tube was used to perform CD4 T lymphocyte typing using flow cytometry on Becton–Dickinson’s “FACS Calibur” according to the manufacturer’s instructions (https://www.bdbiosciences.com/documents/BD_FACSCalibur_Brochure.pdf); the full blood count was performed on the “Mindray BC 3000 plus” automated system as per the manufacturer’s instructions (https://www.mindray.com/en/product/BC-3000Plus.html) and the malaria diagnosis was done by fluorescence microscopy test as per the manufacturer’s instructions (http://www.cyto.purdue.edu/cdroms/cyto10a/sponsors/media/partec/cyscope.pdf). The reliability and accuracy of the results were ensured by the systematic use of standard operational procedures and quality control panels.

Every potential case of thrombocytopenia from full blood count was confirmed by an observation of the blood smear. Thrombocytopenia was defined as a platelet count below 150 × 10^3^/mm^3^ [3], categorized as mild (50,000–149,999 platelets/μL), moderate (20,000–49,999) and severe < 20,000.

#### Statistical analysis

Data were recorded in an Excel spread sheet, and double-checked for data cleaning. The cleaned dataset was then analysed using the software Graph Pad prism version 6. The coefficient of correlation (r) was used to determine the existing relationships between the quantitative variables using the Spearman algorithm. Categorical data were analysed using the Mann–Whitney U test; and the significance threshold for statistical tests was set at 0.05. A linear regression analysis model was used to determinate the association between dependent variable (blood platelet count) and the independent variables. The independent variables included in the model had a p-value ≤ 0.2 with the dependent variable in bivariate analysis.

### Results

#### Characteristics of the study population

A total of 348 people were recruited, but only 310 were eligible and retained. Of the 38 people excluded, 29 had a plasmodium infection and the remaining 09 had hepatic infections (7 Hepatitis B; 2 Hepatitis C). Our study population consisted of 121 (39.03%) males and 189 (60.96%) females giving a female to male ratio of 1.6:1. Participants ranged from 5 to 96 years with a median age of 40 years [IQR: 33–49] (Table 1).

**Table 1:** Socio-demographic data and blood platelet distribution

Variables	Frequency	Percentage (%)
Sex		
Female	189	60.96
Male	121	39.03
Age in years		
0–11	14	4.5
12–19	15	4.8
20–35	74	23.9
36–50	141	45.5
> 50	66	21.3
Blood platelets distribution		
Severe thrombocytopenia < 20,000	0	0
Moderate thrombocytopenia [20,000–50,000[	5	1.61
Mild thrombocytopenia [50,000–150,000[	54	17.42
Normal platelet count [150,000–450,000[	250	80.65
Thrombocytosis > 450,000	1	0.32

#### Blood platelet levels in PLHIV

The prevalence of thrombocytopenia was 19.03% (59/310) with a predominance of mild thrombocytopenia 17.42% (54/310) (Table 1). The mean blood platelet count was 217.64 ± 77.09 and ranged from 34.000 to 466.000/μL.

#### CD4 lymphocyte count and blood platelet count

According to CD4 T lymphocytes, mild thrombocytopenia was 33.33% (17/51) among those with severe immunodeficiency, with a statistically significant difference (p = 0.003) as compared to those with higher CD4. Furthermore, a weak positive and significant correlation was found between CD4 count and platelet count, r = 0.21 (Table 2).

**Table 2:** Blood platelet count according to CD4 lymphocyte and Viral load

Variables	Blood platelet count					Correlation coefficient	P-value
	< 20,000	[20,000–50,000[	[50,000–150,000[	[150,000–450,000[	> 450,000		
CD4 lymphocyte count						r = 0.21	< 0.001
< 200	0 (0.0%)	2 (3.9%)	15 (29.4%)	34 (66.7%)	0 (0.0%)		
[200–300[	0 (0.0%)	1 (2.8%)	5 (13.9%)	30 (83.3%)	0 (0.0%)		
[300–500[	0 (0.0%)	1 (1.2%)	13 (15.9%)	68 (82.9%)	0 (0.0%)		
> 500	0 (0.0%)	1 (0.7%)	21 (14.9%)	118 (83.7%)	1 (0.7%)		
Viral load						r = − 0.12	0.03
Non detectable	0 (0.0%)	1 (0.8%)	19 (14.6%)	110 (84.6%)	0 (0.0%)		
1.60–2.99	0 (0.0%)	0 (0.0%)	12 (11.9%)	88 (87.1%)	1 (1.0%)		
> 3.00	0 (0.0%)	4 (5.1%)	23 (29.1%)	52(65.9%)	0(0.0%)		

#### HIV viral load and platelet count

Up to 34.1% (27/79) of thrombocytopenic patients were on viral load > 3log_10,_ a significant higher burden as compared to those with low-level viremia, p = 0.037 (Table 2). Furthermore, a weak negative correlation was found between platelet count and viral load; r = − 0.12.

#### ART-exposure and blood platelet count

Among naïve PLHIV, 64.6% (42/65) of cases of thrombocytopenia were observed compared to 6.9% (17/245) in treated patients. Depending on the type of ART, 28.9% (11/38) of Thrombopenia patients were observed among patients on AZT-containing HAART compared to 2.9% (6/207) non-AZT containing HAART, p < 0.001 (Table 3).

**Table 3:** Thrombocytopenia according to Sex, Age and ART

Variables	Thrombocytopenia		p-value
	Yes	No	
Sex			0.007
Female	27 (14.3%)	162 (85.7%)	
Male	32 (26.4%)	89 (73.6%)	
Age			0.73
0–11	2 (14.3%)	12 (85.7%)	
12–19	2 (13.3%)	13 (86.7%)	
20–35	13 (17.6%)	61 (82.4%)	
36–50	28 (19.9%)	113 (80.1%)	
> 50	14 (21.2%)	52 (78.8%)	
ART exposure			< 0.001
On ART	17 (6.9%)	228 (93.0%)	
Naive to ART	42 (64.6%)	23 (35.4%)	
AZT exposure			< 0.001
Treatment with AZT	11 (28.9%)	27 (71.1%)	
Treatment without AZT	6 (2.9%)	201 (97.1%)	

#### Adjusting for sex, ART exposure, viral load and CD4 to blood platelets counts

After adjusting for sex, ART, viral load and CD4, Viral load and ART exposure were significantly associated with decreased risk of thrombocytopenia (p < 0.001).

### Discussion

The aim of our study was to assess the burden of thrombocytopenia according to antiretroviral therapy (ART), viremia and CD4-lymphocyte count of PLHIV in view of limiting the occurrence of this haematological disorder and optimizing the management of PLHIV in Yaoundé, Cameroon.

Our study population concerned all HIV positive people followed up in one of the health facilities in Yaoundé with a predominance of women (60.96%). This could be explained by a number of factors that promote the biological vulnerability of women to HIV [4]. Indeed, the female sex has a higher biological receptivity to HIV than the male sex [4]. Our results are consistent with the studies of Kouanfack et al. in 2010 and Essomba et al. in 2015 carried out in Cameroon, which obtained a female predominance of 70.6% and 66.3% respectively [5, 6].

The median age of our study population was 40 years. This could be explained by the fact that HIV infection attacks young people who are sexually active, this being the case of the Yaoundé population. This median age is close to that obtained in Cameroon by Essomba et al. [6] in 2015 who found 43 years.

In our study, 19.03% of subjects had thrombocytopenia; this result is different from that obtained by Tene et al. [7] in 2014 in Yaoundé Cameroon, which reported a prevalence of 13.67%. This can be explained by the fact that their study population of 139 participants was unrepresentative. On the other hand, our result is close to that obtained by Taremwa et al. [8] in southwestern Uganda in 2015, in which 17.4% was obtained and the 20% obtained by Alaei et al. [9] In Iran in 2000.

Depending on the severity of thrombocytopenia, the most predominant form was mild thrombocytopenia. The absence of severe thrombocytopenia can be explained by the fact that most of the patients followed up in Yaoundé who came for biological monitoring at the CIRCB were not in absolute emergency, and because most (79.0%) of these patients were on treatment. Furthermore it has been shown that an appropriate antiretroviral therapy is the most important step to fight thrombocytopenia [10].

Concerning severe immunodeficiency, 33.3% had thrombocytopenia. There was a positive and significant correlation between blood platelet count and CD4 count, which implies that a decrease in CD4 cell count may be accompanied by a decrease in blood platelet count. Taremwa and colleagues [8] in Uganda have also found a significant association between blood platelet count and CD4 T cell lymphocyte count (p < 0.05).

Virological failure (viral load > 3log_10_) was present in 45.7% of thrombocytopenic patients. There was a negative and significant correlation between blood platelet count and plasma viral load, which could be explained by the fact that an increase in viral activity could be accompanied by a decrease in blood platelet count [11]. This result is in perfect agreement with that of O’Bryan et al. [10] in the United States of America in 2015 who also found an inverse correlation between platelet count and HIV viral load. It has been described during HIV infection that thrombocytopenia can occur by various mechanisms including bone marrow destruction induced by viral activity, antiretroviral therapy or immunological factors [11]. In Cameroon Mbanya et al. [12, 13] in 2002 identified HIV as a cause of thrombocytopenia.

In our study, 6.9% of people on antiretroviral therapy had thrombocytopenia. This result is less than the 13% obtained by Taremwa and colleagues in Uganda [8], and higher than the 4.1% obtained by Wondinemeh et al. in Ethiopia [12, 13]. 28.9% (11/38) of thrombocytopenic ART-treated patients were on AZT-containing therapies. This result could certainly suggest the involvement of AZT in the occurrence of drug induced thrombocytopenia [14]. On the other hand, 37% of antiretroviral-naïve patients had thrombocytopenia. This result is not in agreement with that obtained by Wankah et al. [15] at the Yaoundé teaching Hospital, which obtained 27.1%. This large difference in the frequency of thrombocytopenia among HIV patients in Yaoundé under ART compared to those naïve to ART reflects the findings by O’Bryan et al. [10] in the United States who showed decreased prevalence of thrombocytopenia in an HIV infected population as we moved from the pre-therapeutic periods to during early therapy and then to therapeutic periods with highly active antiretroviral drugs (HAART).

After adjusting for sex, ART, viral load and CD4, viral load and ART exposure were significantly associated with decreased risk of thrombocytopenia. This result justifies the major implication and the protective effect of ART among risk of thrombocytopenia [16].

### Conclusion

In this population of PLHIV followed routinely in Cameroon, thrombocytopenia affects less than a quarter with the majority having only mild thrombocytopenia. Thrombocytopenia occurs especially among ART-naïve, AZT-containing regimens, high viremia and severe immune-compromised patients. In addition, ART coverage appears as an independent factor in preventing the occurrence of thrombocytopenia, further justifying universal treatment for all PLHIV. However, an alternate therapeutic approach is needed for AZT-sparing treatment combinations in countries with similar features like Cameroon.

## Limitations

The main limitation of our study was that we couldn’t determine the origin of platelet deficiency as being peripheral or central and also did not use blood platelet reference values specific to our context that could certainly give us another view of blood platelet deficiency trends. It would be interesting in future studies to determine the platelet reference values specific to our context and to enrol more children and older people in order to better appreciate the category most vulnerable to this defect among PLHIV.

## Data Availability

The dataset is available from the corresponding author on reasonable request.

## Abbreviations

ARV: antiretrovirals
AIDS: Acquired Immunodeficiency Syndrome
HIV: Human Immunodeficiency Virus
PLHIV: people living with HIV
TARV: antiretroviral therapy
CIRCB: “Chantal Biya” International Reference Center for research on HIV/AIDS prevention and management
CHU: Yaoundé Hospital and University Center
